# Effect of ultrasound pre-treatments before enzymatic hydrolysis on physicochemical, nutritional and functional properties of insoluble protein fractions obtained from Atlantic mackerel side streams

**DOI:** 10.1186/s40643-025-00931-3

**Published:** 2025-08-23

**Authors:** Janna Cropotova, Kristine Kvangarsnes, Elissavet Kotsoni, Revilija Mozuraityte, Inger Beate Standal, Amélie Le Gall, Turid Rustad

**Affiliations:** 1 Department of Biological Sciences Ålesund, Norwegian University of Science and Technology, Larsgårdsvegen 4, Ålesund, 6025 Norway; 2 SINTEF Ocean, Brattørkaia 17C, Trondheim, NO-7010 Norway; 3 L’institut Agro Dijon, 26 bd du Docteur Petitjean, Dijon Cedex, 21079 France; 4 Department of Biotechnology and Food Science, Norwegian University of Science and Technology, Sem Saelandsvei 6/8, Trondheim, 7491 Norway

**Keywords:** Insoluble protein, Ultrasound treatment, Enzymatic hydrolysis, Atlantic mackerel, ^31^P-NMR, ^1^H NMR, Oxidation

## Abstract

This study investigated the impact of ultrasound (US) pre-treatment before enzymatic hydrolysis on physicochemical, nutritional, and functional properties of insoluble protein fractions obtained from fish side streams after filleting of Atlantic mackerel (heads, backbones, fins, tails and trimmings). Four fractions were analyzed: a control without US-treatment and US-treated samples at 300 W, 450 W, and 600 W. The results showed that ultrasonication significantly increased total protein content in insoluble protein fraction after the treatment at 300 W. Additionally, salt-soluble proteins increased significantly in 450 W and 600 W samples, while water-soluble proteins increased after US-treatment at 600 W, suggesting that higher US-intensities promoted protein bond breakdown and exposure of hydrophilic amino acid groups. The degree of hydrolysis increased significantly in 450 W sample due to cavitation effect of ultrasound. Total thiols decreased in water-soluble proteins after US-treatment at 300 W and 450 W, as well as in all salt-soluble proteins compared to control due to unfolding of proteins and breakage of disulfide bonds. No significant differences were detected in lipid oxidation markers (TBARS and ¹H NMR aldehyde profile). From the nutritional perspective, the nutritional indexes of lipid quality AI (atherogenicity index), TI (thrombogenicity index), and HH (hypocholesterolemic fatty acid ratio) were favorable for all insoluble protein fractions. US-treatment enhanced the hypocholesterolemic profile of insoluble protein fractions by lowering their HH index. P-NMR lipid profiling identified PC and PC-ether as the predominant phospholipids (70–74% of total PL content). No significant differences were found in color parameters (L, a, b*) between control and US-treated samples at 300 W and 450 W.

**Graphical abstract:**

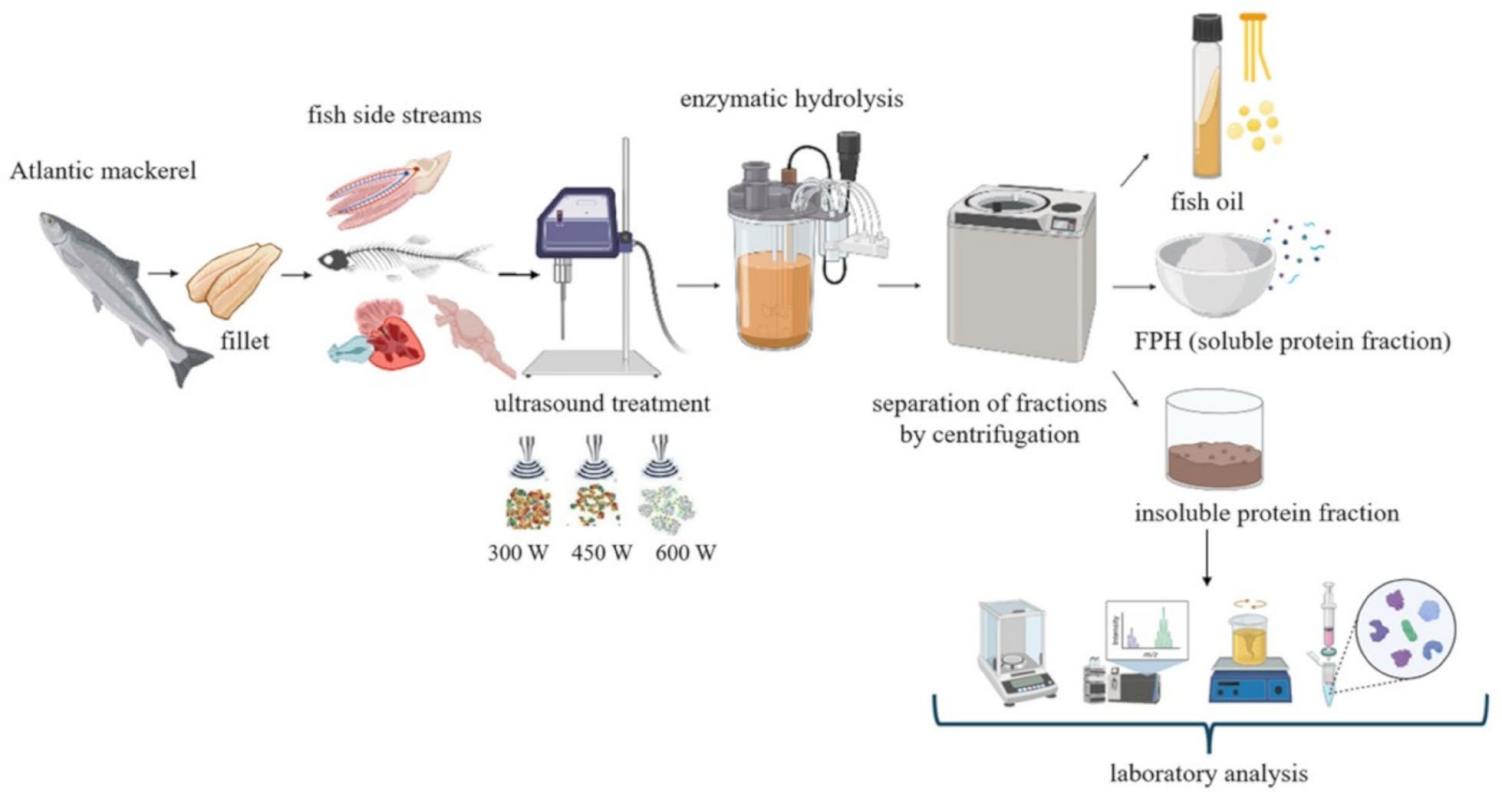

## Introduction

At the present time, the global food industry is facing major challenges as it endeavors to feed the world population exceeding 7 billion people (Barclay and Haschke, 2015). Along with the continuously increasing world population, the pressure on the livestock feed production industry and its environmental impact is also rising (Maja and Ayano 2021). Taking into account that about 60% of the global protein production is used to provide feed to cattle and chicken farms (Salter and Lopez-Viso 2021), which are also growing in number, it is absolutely necessary to find new sources of high-quality protein ingredients. In terms of protein quality, seafood, and particularly fish, is one of the best sources of complete protein, providing all essential amino acids in adequate proportions, with a digestibility rate of around 98.3–98.8% (Pyz-Łukasik et al. 2020). Nevertheless, only 30–40% of the total fish production is used for direct human consumption, and the remaining 60–70% of the residual material are normally discarded or used for low value applications such as fish meal or oil (Siddiqui et al. 2023). Fish discards are a rich source of essential nutrients (vitamins, polyunsaturated fatty acids, minerals, etc.) as well as complete protein containing all essential amino acids (Pyz-Łukasik et al. 2020). These nutrients can be recovered and utilized in both human and animal diets, offering a functional and nutritious product with health-promoting properties (Bartolomei et al. 2023; Cropotova et al. 2024a; Kotsoni et al. 2024a).

To improve the utilization of fish side streams and by-products, new technological solutions and methods for processing are required. With innovative technological solutions and advanced processing methods, such as high pressure processing (HPP) (Kotsoni et al. 2024a), microwave-assisted extraction (MAE) (Al Khawli et al. 2019; Dong et al. 2024), ultrasonication (US) (Cropotova et al., 2024), supercritical fluid extraction (SFE) (Al Khawli et al. 2019; Dong et al. 2024), there is a big potential in full utilization of fish side streams and marine residual biomass. One such innovative technological solution is ultrasound pre-treatment coupled with enzymatic hydrolysis (Cropotova et al. 2024b). Enzymatic hydrolysis is the process of splitting protein molecules into smaller components using enzymes (e.g. proteases) and water (Cruz-Casas et al. 2021). This process makes it possible to valorize fish side streams by recovering high-quality soluble protein compounds called fish protein hydrolysates (FPH). FPHs represent a mix of soluble peptides of different sizes with high nutritional value and bioactive properties (Cruz-Casas et al. 2021). However, during enzymatic hydrolysis, fish side streams are separated into different fractions, as follows: lipid fraction (if fatty fish is used), water-soluble fraction and insoluble (solid) fraction (Kotsoni et al. 2024b). The water-soluble fraction consists of soluble peptides of different sizes and other water-soluble compounds (vitamins, antioxidants, etc.). This is a valuable fraction used to obtain peptide ingredients for the food industry, pet food, nutraceuticals, cosmetics and pharma (Cropotova et al. 2024a). The lipid phase consists of fish oil and fat-soluble compounds (vitamins, phospholipids, etc.) (Kvangarsnes et al. 2021a, b). The insoluble fraction consists of solids (bones and/or shells) and insoluble protein compounds (Kotsoni et al. 2024b). This fraction is normally used to make fish meal, bone/shell meal, or fertilizer. Thus, enzymatic hydrolysis of fish side streams represents a fast, safe and chemical-free method for the effective separation of peptide fractions (FPH), bones and fish oils from complex matrices. Previously, we have shown chemical and proximate compositions of the different fractions obtained after enzymatic hydrolysis of Atlantic mackerel (Cropotova et al. 2024b), rainbow trout and Atlantic salmon (Kvangarsnes et al. 2021a, b; Kotsoni et al. 2024b), with the help of advanced non-thermal technologies. The majority of the published studies on enzymatic hydrolysis of fish have mostly been focusing on the quality and functional properties of fish oil and FPH, with limited research conducted on insoluble protein fraction (sediments). However, since the solid fraction also contains essential nutrients such as phospholipids rich in omega-3 fatty acids, indispensable amino acids including tryptophan, and trace elements like selenium, iron and zinc (Liaset and Espe, 2008), uplifting its quality, functional and nutritional properties could help finding new areas of added-value applications. The residual insoluble fraction obtained after enzymatic hydrolysis consists mostly of hydrophobic peptides that aggregate and precipitate out of the hydrolysate, as well as proteins that resisted enzymatic breakdown, including collagen, elastin and other connective tissue protein molecules if fish skin and bones were used (Liaset et al. 2003). Although less explored than soluble hydrolysates, the insoluble protein fraction can be used in fish and animal feed formulations or incorporated into different foods for texture enhancement (Kechaou et al. 2009). However, this protein fraction can have a strong bitter taste due to a high amount of hydrophobic peptides and free amino acids (Liaset et al. 2003), which may limit its sensory appeal for end users.

Thus, utilizing all fractions, including the insoluble protein fraction obtained after enzymatic hydrolysis of fish side streams for higher value applications such as pet food or nutraceuticals, could become a resource-saving and sustainable approach.

The main aim of this study was to investigate the effect of US-treatment before enzymatic hydrolysis on physicochemical, nutritional and functional properties of the insoluble protein fraction obtained after the precipitation step following the centrifugation of the hydrolysate mixture of Atlantic mackerel side streams.

## Materials and methods

### Ultrasound pre-treatment and enzymatic hydrolysis of mackerel side streams

Fresh side streams (heads, backbones, fins, tails and trimmings) of Atlantic mackerel (*Scomber scombrus*) obtained after filleting of the fish at a local fish processing facility (Fosnavåg, Norway), were delivered to NTNU (Ålesund, Norway) in insulated containers filled with ice in October 2023. The fish side streams were minced right after the delivery using a mincer with 4.5 mm hole size (Hobart A 200 N), divided into batches of 1 kg, and immediately frozen and stored at -80 °C until further ultrasound pre-treatment and enzymatic hydrolysis. The proximate composition of the minced mackerel side streams is presented in Cropotova et al. (2024b). Due to the large volumes of mackerel harvested in a short period, freezing is commonly used as a preservation method, either before filleting, or to preserve side streams during the fresh fish season. Freezing of fish side streams before enzymatic hydrolysis is a common practice in fish processing industry (Cropotova et al. 2024b; Kotsoni et al. 2024b). It results in ice crystal formation inside fish flesh, which can lead to disruption of cellular membranes and protein structures, including the denaturation of myofibrillar proteins, reducing their solubility and functional properties (Sapatinha et al. 2025). However, Alcalase^®^ enzyme used in the present study, has shown good hydrolytic efficiency on frozen fish co-products (Sapatinha et al. 2025), and thus the freezing step was not regarded as a technical drawback.

On the day of experiment, the prepared mackerel mince samples were subjected to ultrasound pre-treatment at the ultrasonic power of 300 W, 450 W and 600 W with a 20 kHz probe (Sonics & Materials Inc., Danbury, CT., USA, model: VCX 1500) for 10 min. The probe has a vibrating titanium tip of 1.2 cm which was immersed into the mixture of mackerel side streams and water (ratio 1:1 w/w, in 400 mL) followed by its irradiation with an ultrasonic wave directly from the horn tip. Samples were treated for 10 min with intervals of 5 s passive (rest) and active (treatment) phase each. The temperature of the mixture was controlled during the sonication, and the value was 50 ± 2 °C.

Right after the ultrasonication, enzymatic hydrolysis was performed on both the experimental (US-treated) and control (untreated) mackerel samples in 4-litre closed glass bioreactors placed in a water bath at 52 °C. Warm (50 ± 2 °C) distilled water was added to the control fish mince in a 1:1 w/w ratio to achieve the same temperature as ultrasonicated samples. The US-treated experimental samples had already been added water in the ratio of 1:1 w/w, and the temperature of the mixture was 50 ± 2 °C right after the ultrasonication. The mixtures were stirred at 150 rpm with an overhead stirrer. When the temperature of the mixtures was 50 °C, Alcalase^®^ enzyme (Sigma-Aldrich, Germany) was added in all bioreactors at the level of 0.1% (w/w) of the raw material weight. After 60 min of hydrolysis, bones were removed by filtering the hydrolysate through a sieve before the enzymes were inactivated by heating up to 90 °C for 10 min in a microwave oven. The mixture was cooled down up to 30 °C before being transferred to one litre centrifugation bottles and then centrifuged at 4100 g at 4 °C for 30 min. The liquid fractions (lipids and water-soluble proteins) were separated from the insoluble fraction. The water-soluble protein phase representing fish protein hydrolysate (FPH) and insoluble protein fraction were further dried in the laboratory vacuum freeze-dryer (Labconco Freezone Console 12 L Freeze Dry System). The freeze-dried insoluble protein fraction collected after each hydrolysis experiment was used for physicochemical analysis.

### Proximate composition analysis

Water content was determined gravimetrically after drying at 105 °C for 24 h. Ash content was determined by incineration to constant weight at 550 °C (AOAC 2000). Lipid content was determined by the Bligh & Dyer (B&D) method (Bligh and Dyer 1959) using a binary mixture of chloroform and methanol diluted with distilled water as an extraction medium, as described by Mozuraityte et al. (2021). The amount of protein (in g) in the samples was determined according to the Kjeldahl method as described by Cropotova et al. (2024a).

### Protein solubility of mackerel fish mince and insoluble protein fraction

Water-soluble (sarcoplasmic) and salt-soluble (myofibrillar) proteins were determined in the insoluble protein fraction according to the method previously described by Kvangarsnes et al. (2021a, b), as follows. The protein extracts were prepared by dissolving 0.1 g of each sample in 10 mL of distilled water. The solutions were homogenized and centrifuged. The supernatant was filtered through glass wool into a 100 ml volumetric bottle, and the volume was made up to 100 ml with more buffer. This was the water-soluble protein fraction. To obtain salt-soluble protein fraction, 80 ml 0.05 M phosphate buffer with 0.6 M KCl were added to the centrifuge bottle, shaken a little to get dissolve the precipitate high on the walls, homogenized with Ultra Turrax for 30 s and centrifuged at 8000 g at 4 °C for 20 min. The resulting supernatant represented the salt-soluble protein fraction. Water- and salt-soluble proteins were determined in triplicates by using the Lowry method (Lowry et al., 1951). Bovine serum albumin (BSA) was used to prepare a standard curve. The absorbance of the incubated standards and samples was determined using a SpectraMax ix3 microplate reader (Molecular Devices, USA) at a wavelength of 750 nm. The analyses were run in triplicate and the mean value ± SD was calculated.

Protein solubility was calculated from the following formula (1):

1 $$\:Protein\:solubility=\:\frac{soluble\:proteins\:in\:extract}{total\:protein\:content}\:x\:100$$

### Degree of hydrolysis

The degree of hydrolysis (DH) of insoluble protein fraction was determined to see the amount of hydrolysed peptides that went into the sediments. The analysis was performed by formol titration and DH was calculated as the proportion (%) of free amino groups with regard to the total protein content in the sample. 1.5 g of an insoluble protein sample was weighed into a beaker and filled up to 50 g with distilled water. The pH was adjusted to 7.0 using 0.1 M NaOH and then 10 ml of 9% w/w formaldehyde with a pH of 8.5 was added. The beaker was covered with aluminium foil and stirred for 5 min. For the titration, a TITROLINE 7000 automatic titrator (SI Analytics, Xylem Analytics Germany Sales GmbH & Co. KG, Germany), was used. The titrator was rinsed 3 times before starting the titration. Furthermore, the titration was set to pH 8.5 with stopping automatically when reaching a pH of 8.5. The samples were titrated with 0.1 M NaOH and the used amount of NaOH was recorded. Degree of hydrolysis was further determined as described by Cropotova et al. (2024).

### Fatty acid composition of the lipid part in insoluble protein fraction

The fatty acid composition of the lipid phase in the insoluble protein fraction was determined following the official method displayed in AOCS (1998). Approximately 25 mg of oil was dissolved in 1.5 mL of 0.5 N NaOH under heat at 100 °C for 5 min. After cooling, the mixture was esterified by adding 2 mL of BF3/methanol, followed by heating at 100 ◦C for 30 min. Upon cooling, 1 mL of isooctane was added, and the tubes were vortexed. Subsequently, 5 mL of saturated NaCl solution was added. The resulting isooctane layer was then transferred into vials and analyzed further by gas chromatography (GC). The gas chromatograph (Perken Elmer Autosystem XL) was equipped with a CB-WAX 52CB capillary column (25 m × 0.25 mm) and a flame ionization detector. The injector and detector temperatures were kept at 220 °C and 250 °C, respectively. The oven temperature was held at 90 °C for 1 min. Then, the temperature was raised to 150 °C at a rate of 45 °C/min, followed by a further increase to 220 °C at a rate of 3.5 °C/min, where it was held for 2 min. Hydrogen was used as the gas carrier. The results were expressed as mean ± SD (*n* = 3) % area fatty acids in GC.

#### Nutritional quality of the lipid part

To assess nutritional quality of the lipids found in the insoluble protein fraction, the data from the fatty acid composition analysis was used to calculate the following lipid nutritional quality indexes: ratio of ω-6 to ω-3 fatty acids, atherogenicity index (AI), thrombogenicity index (TI), and hypocholesterolemic fatty acid ratio (HH), as suggested by Chen and Liu (2020).

AI represents the relationship between pro-atherogenic (primary saturated fatty) and anti-atherogenic (primary unsaturated) fatty acids (Dave et al. 2024). This index was first developed by Ulbritcht and Southgate (1991) to characterize the atherogenic potential of fatty acids and displays the relationship between pro-atherogenic fatty acids promoting the adhesion of lipids to the cells of circulatory systems and anti-atherogenic fatty acids inhibiting the aggregation of plaque and reducing the levels of cholesterol, phospholipids and esterified fatty acids (Senso et al. 2007). The lower AI index reduces the risk micro- and macrocoronary diseases (Senso et al. 2007; Garaffo et al. 2011). AI index is calculated according to the following Eq. (2) using % of total fatty acids in calculations (Senso et al. 2007):

2 $$\:AI=\frac{(C12:0+4*C14:0+C16:0)}{(\varSigma\:MUFAs+PUFA(n6)+PUFA(n3\left)\right)}$$

TI displays the relationship between pro-thrombogenetic (saturated) and anti-thrombogenetic (MUFAs, PUFA(n-6), and PUFA(n-3) fatty acids) fatty acids (Senso et al. 2007; Dave et al. 2024), showing the risk of clot formation in the blood vessels. Thus, the consumption of lipid-rich foods with a lower TI is beneficial for cardiovascular health (Chen and Liu 2020). TI determines according to the following formula, using % total fatty acids in calculations (3):

3 $$\begin{aligned} & TI \\ & =\frac{(C14:0+C16:0+C18:0)}{\left[\begin{matrix}(0.5*\varSigma\:MUFAs)+(0.5*PUFA(n6))+\\(3*PUFA(n3))+\raisebox{1ex}{$PUFA(n3)$}\!\left/\:\!\raisebox{-1ex}{$PUFA(n6)$}\right.\end{matrix} \right]} \end{aligned}$$

The HH ratio is associated with cholesterol metabolism (Łuczyńska et al. 2023; Dave et al. 2024) and is calculated according to the following formula (4):

4 $$\begin{aligned} & HH \\ &= \frac{\left[\begin{matrix}C18:1(n9)+C18:2(n6)+C18:3(n3)+\\C20:5(n3)+C22:5(n3)+C22:6(n3)\end{matrix}\right]}{C14:0+C16:0}\end{aligned}$$

### Amino acid composition in the insoluble protein fraction

The amino acid composition in the insoluble protein fraction was assessed following the methodology outlined by Blackburn (1978). Prior to analysis, 50 mg of each sample underwent hydrolysis at 105℃ for 22 h using 1 mL of 6 M HCl. Afterward, neutralization was carried out by adding NaOH until the pH reached 7. The samples were then filtered, and their volume was adjusted to 10 mL with distilled water. Subsequently, appropriate dilutions were made, followed by filtration, after which the samples were transferred into vials for further evaluation using high-pressure liquid chromatography (VHPLC Dionex Ultimate 3000, Thermo Fischer Scientific Inc., USA) fitted with a Nova Pak C18 column (WatersTM, USA). The final results were presented as mean ± SD (*n* = 3) in mg/g protein.

### Lipid and protein oxidation parameters in the insoluble protein fraction

Lipid oxidation of the lipid part in the insoluble protein fraction was assessed in terms of secondary lipid oxidation products through determination of Thiobarbituric acid reactive substances (TBARS) according to Ke and Woyewoda (1979) as described by Kvangarsnes et al. (2021a, b). For determination of TBARS, 200 µl of chloroform phase from the Bligh and Dyer extraction of lipids from the insoluble protein fraction was taken. The results were expressed as mg MDA/ kg oil.

Protein oxidation of the insoluble protein fraction was assessed in terms of total thiol groups determined according to Ellman (1959) and Kvangarsnes et al. (2023). To 0.1 ml of the water-soluble and salt-soluble extract of the insoluble protein fraction and blank (distilled water), 0.8 ml of 8 M urea and 0.1 ml of DTNB were added. The samples were mixed, incubated at room temperature for 30 min, and centrifuged for 3 min at 11 000 g at room temperature. The absorbance was measured spectrophotometrically with Shimadzu UV-1800 UV/visible scanning spectrophotometer (Shimadzu Europa GmbH, Germany) at 412 nm with the blank as reference. The thiol content was calculated in both water (sarcoplasmic) and salt-soluble (myofibrillar) protein extracts using a molar extinction coefficient of 14,290 M^− 1^ cm^− 1^. The results were expressed as nmol/mg protein.

### NMR profile of lipids in the insoluble protein fraction

Comparative analysis of composition of lipids extracted from the insoluble protein fractions (sediments) were conducted using ^31^P NMR and ^1^H NMR profiling.

^31^P NMR – was NMR was run on a Bruker 400 MHz Avance Neo instrument (Bruker Biospin GmbH, Rheinstetten, Germany) at the NV Faculty, NTNU. A procedure with CsEDTA washing to remove cations in the sample prior to 31P NMR analyses was employed (Menezes and Glonek, 1988, Monakhova et al., 2018). The Cs-EDTA solution was prepared as described by Monakhova et al. (2018), by weighing 1.45 g of EDTA and 3 g of Cs2CO_3_ and dissolving in 25 mL distilled water.

The lipid extract sample was added deuterated chloroform (CDCL3) (0.65 mL), 0.65 mL methanol (type used in HPLC) containing triphenyl phosphate (as internal standard), and 0.65mL CsEDTA solution. The mixture was stirred for approximately 20 min, prior to centrifugation. Two phases formed, and the lower organic phase was transferred to the 5 mm NMR tubes. Acquisition parameters were: pulse program zgig, time domain 262,144 k, spectral width 81 ppm, acquisition time 9.96 s, relaxation delay 2.0 s, number of scans 64, dummy scans 4. One zero filling and a line broading of 2 Hz were applied. Assignments were made according to spectra of pure compounds, and literature values (Monakhova et al., Menezes and Glonek et al., Burri et al. 2016; Standal et al. 2024). The analyses were performed in parallel.

The phospholipid (PL) contents were reported as weight% of extracted lipids, while individual phospholipid species were expressed as weight% of total quantified phospholipids. Calculations were done as described in Standal et al. (2024), and by using the average molecular weights of phosphatidylcholine (PC) and its ether, lysophosphatidylcholine (LPC), phosphatidylinositol (PI) and phosphatidylethanolamine (PE) (PC + PC ether: 790 g/moles, PI: 907 g/moles, LPC 535 g/mole, PE: 770 g/mole (Monakhova et al., 2018).

^1^H NMR was run on a Bruker 600 MHz Avance III HD, equipped with a 5-mm cryoprobe, at the same location. The lipid extract sample (100 mg) was added 0.6 mL CDCL3 containing Trimethylsilane (TMS) as chemical shift reference and transferred to 5 mm NMR tubes. The following acquisition parameters were used: pulse program zg30, time domain 64k, spectral width 18.03 ppm, acquisition time 3.03 s, relaxation delay 7.0 s, number of scans 128, and dummy scans 4. Zero filling and exponential line broadening (0.30 Hz) was applied before Fourier transform. Evaluation of the content of triacylglycerols (TAGs) and diacylglycerols (DAGs) were made by integrating corresponding peaks in the glycerol region (see e.g. Standal et al. 2024), in addition to peaks from the methyl end of fatty acids. Results were expressed as mole % of total fatty acids. Peaks from aldehydes were identified based on literature values (Skiera et al., 2012).

### Colour parameters

Colour parameters of the insoluble protein fraction were determined using a Minolta Chromometer Model CR 400 (Konica Minolta, Japan) calibrated on a white reference plate before use. L* (lightness), a* (redness) and b* (yellowness) were measured on surfaces of insoluble protein fractions in triplicate at a room temperature. The L*, a* and b* parameters of the CIELAB scale were measured according to the lab scale established by Commission Internationale de l’Éclairage (CIE 2001), and the average with standard deviation were calculated.

### Statistical analysis

All results were expressed as the mean ± standard deviation (s.d.), where p-values < 0,05 were considered to be significant. Statistical analyses were performed by one-way ANOVA followed by Tukey’s post-test (Sigma Plot, version 7, USA).

## Results and discussion

### Proximate composition

The proximate composition of mackerel raw material used for the experiment was previously published in Cropotova et al. (2024b), while the proximate composition of insoluble protein fractions obtained after enzymatic hydrolysis with and without different US pre-treatments, is shown in Table 1.

**Table 1:** Proximate composition of insoluble protein fraction obtained after enzymatic hydrolysis of mackerel side streams (wet weight basis). Mean values ± standard deviation is shown. Different letters indicate significant differences (*p* < 0.05)

Parameters	Insoluble protein fractions after enzymatic hydrolysis			
	Control	300 W	450 W	600 W
Total protein content, %	17.0 ± 0.1^a^	18.9 ± 0.1^b^	15.3 ± 0.1^c^	17.5 ± 0.1^a^
Lipid content, %	8.9 ± 1.6^a^	9.8 ± 1.9^a^	9.9 ± 1.8^a^	9.6 ± 2.2^a^
Water content, %	72.8 ± 0.5^a^	70.1 ± 0.3^b^	73.1 ± 0.2^a^	71.8 ± 0.4^ab^
Ash, %	1.4 ± 0.3^a^	1.2 ± 0.4^a^	1.6 ± 0.2^a^	1.2 ± 0.1^a^

As can be seen from Table 1, total protein content increased in experimental samples of insoluble protein fraction after US-treatment at 300 W and 600 W compared to control. However, the significant differences (*p* < 0.05) were observed only for the sample 300 W. The significantly lowest protein content was observed for the sample US-treated at 450 W. The significant decrease in protein content after US-treatment at 450 W compared to all other samples, can be explained by the stronger cavitation effect with an increase in ultrasonic power, enhancing emulsification of proteins and yielding insoluble protein fraction with lower protein content (Cropotova et al. 2024b). This suggestion is supported by the fact that insoluble protein fraction obtained after sonication at 450 W, had the highest lipid content among all other samples (Table 1), and could therefore become more emulsified by the proteins released after the rupture of mackerel mince during US-assisted hydrolysis. Considering the highest lipid content in insoluble protein fraction after US-treatment at 450 W compared to all other samples, we suggest that proteins were emulsified by the higher amount of phospholipids released after the sonication. This hypothesis is in agreement with our previously published study showing the lowest protein content together with the highest lipid content in mackerel FPH after US-treatment at 450 W (Cropotova et al. 2024b). At the same time, there was no significant difference observed between all the samples in terms of total lipid and ash content. According to our hypothesis supported by previous investigations (Cropotova et al. 2024b), lower ultrasonic power of 300 W may enhance the enzyme activity and its access to the substrate. This phenomenon increases the efficiency of enzymatic hydrolysis which results in higher protein yield insoluble protein fractions (Ma et al. 2011). However, application of higher ultrasonic powers (450 W and 600 W) may induce a stronger cavitation effect followed by emulsification of proteins with lipids, which decreases protein content in both soluble and insoluble fractions.

### Soluble proteins

**Fig. 1:**
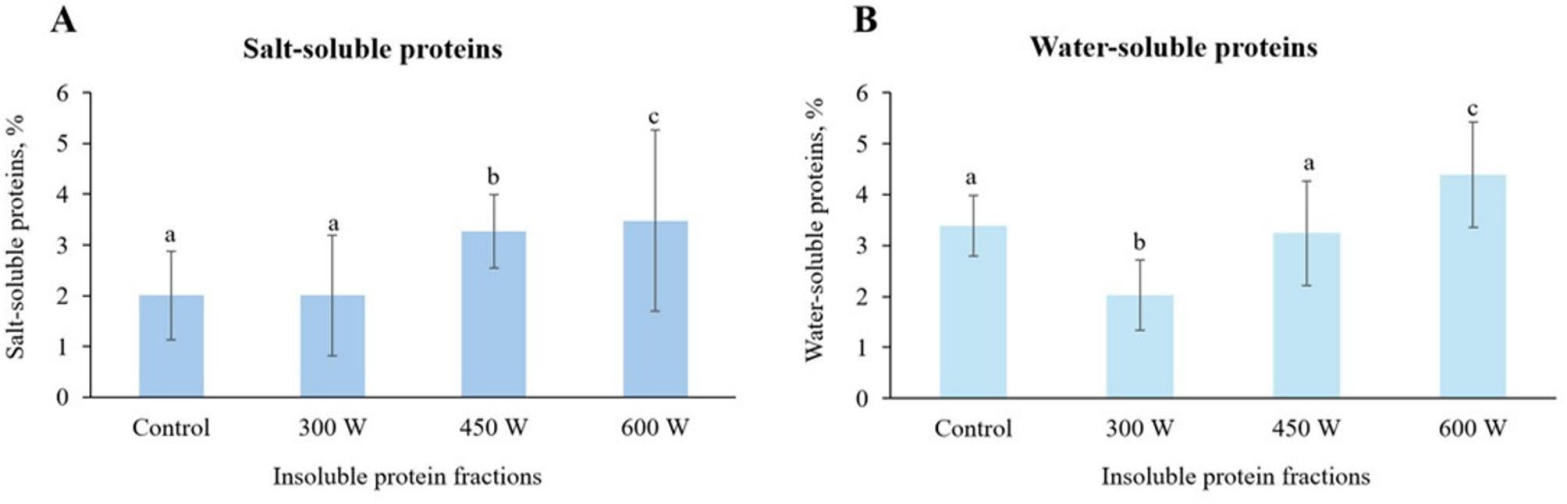
Water- and salt-soluble proteins in insoluble protein fraction, %. Mean value and standard deviation are shown. Different letters indicate significant differences (*p* < 0.05)

According to Fig. 1A, there was a significant (*p* < 0.05) increase in salt-soluble proteins in experimental samples after US-treatment at 450 W and 600 W, and in water-soluble proteins after ultrasonication at 600 W compared to control. One of the possible explanations can be breaking down hydrogen and hydrophobic bonds of proteins as a result of strong cavitation effect, leading to the exposure of hydrophilic groups of amino acid residues, which increases protein solubility (Tang et al., 2021). Thus, US-treatment which generated not only cavitation, but also turbulence, surface electrostatic and thermodynamic effects, caused structural opening and partial protein denaturation already before the enzymatic hydrolysis. This further resulted in the generation of additional negative charges on the protein surface, promoting their hydration (Kornet et al. 2021). With an increase in ultrasonic power, both the cavitation effect and shear force increased, leading to depolymerization of salt-soluble protein aggregates, thereby improving their solubility (Ciuti et al. 2000). Another explanation is the reduction of the particle size of proteins increasing the surface area of proteins contacting with water (Arzeni et al. 2012; Gao et al. 2022). Thus, high power US-treatment can make protein molecules more soluble by disintegrating larger molecular size protein aggregates into smaller ones (Tang et al. 2009). At the same time, US-treatment at 300 W did not affect the amount of salt-soluble proteins (1A), while there was a significant (*p* < 0.05) drop in water-soluble proteins after ultrasonication at this sonic power compared to control (1B). This confirms our previous statement that US-treatment at lower ultrasonic powers enhances efficiency of enzymatic hydrolysis (Ma et al. 2011), which results in higher yield of fish protein hydrolysate (soluble fraction) and subsequently reduces the amount of water-soluble proteins in insoluble protein fraction.

### Degree of hydrolysis

**Fig. 2:**
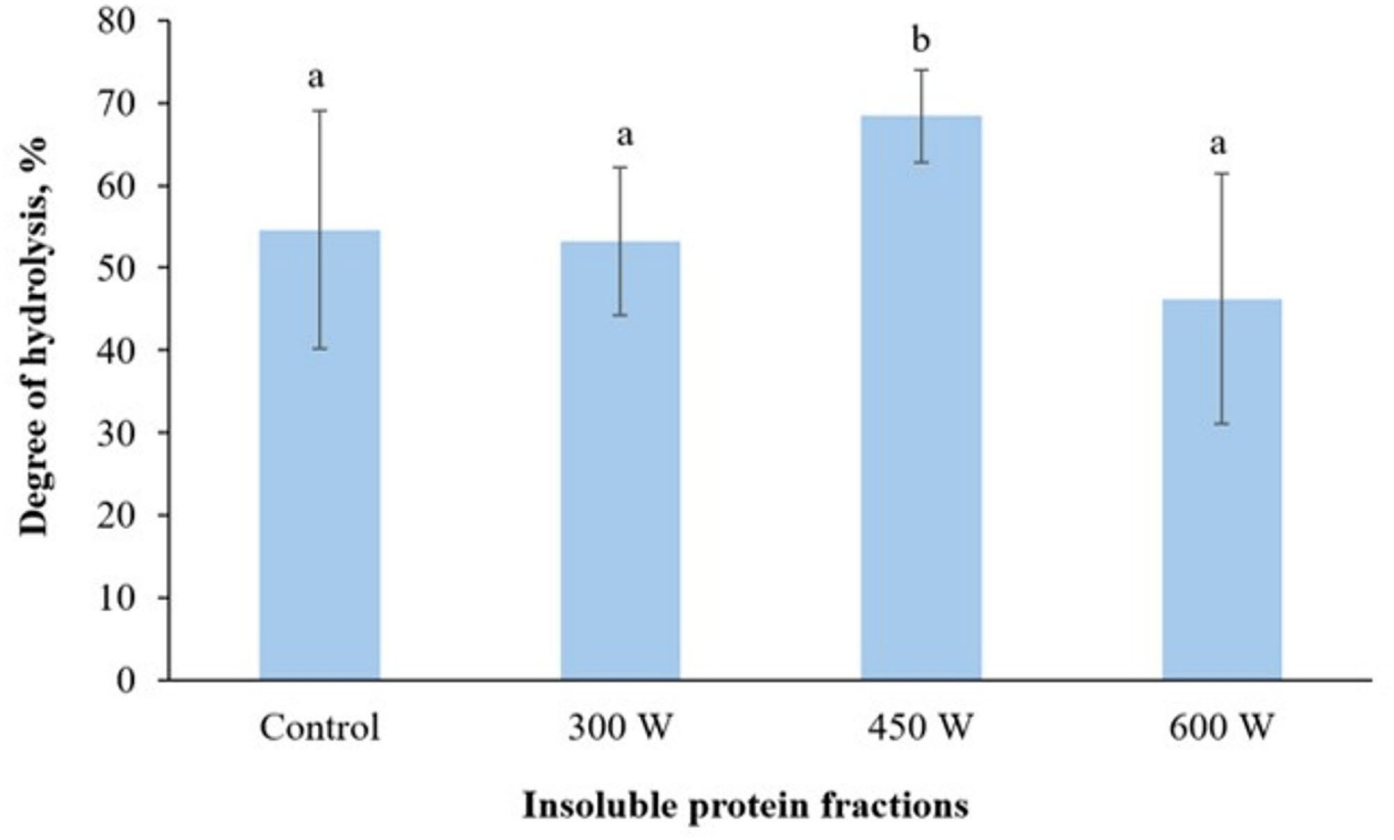
Degree of hydrolysis in insoluble protein fraction. Mean value and standard deviation are shown. Different letters indicate significant differences (*p* < 0.05)

According to the results displayed on Fig. 2, the degree of hydrolysis increased significantly (*p* < 0.05) in the insoluble protein fraction after US-treatment at 450 W compared to control. At the same time, there was no significant difference in the degree of hydrolysis between the sample ultrasonicated at 300 W and the control. This phenomenon supports our previous hypothesis that ultrasonic cavitation effect intensifies breaking hydrogen and hydrophobic bonds of proteins resulting in greater exposure of substrate to enzymes, which enhances enzymatic hydrolysis (Ma et al. 2011; Tang et al., 2021). In addition, there was a slight, but insignificant decrease in degree of hydrolysis for insoluble protein fraction sample obtained after US-treatment at 600 W. This effect can be explained by aggregation and denaturation of proteins due to increased combined cavitation and mechanical oscillation effect at higher US power, altering both the substrate and the enzyme activity (Huang et al. 2017) and resulting in decreased degree of hydrolysis (Umego et al. 2021).

### Fatty acid composition

Table 2 displays fatty acid composition of the lipid phase of the insoluble fraction obtained after enzymatic hydrolysis of mackerel side streams. The fatty acids C16:0, C18:1 (n-9), and C22:6 (n-3), and C22:1 (n11 + 13) were the most abundant across all samples. It was noticed that the amount of palmitic (C16:0) and oleic (C18:1 (n-9) acids increased significantly (*p* < 0.05) in all US-treated samples compared to control. However, the content of docosahexaenoic acid (DHA, C22:6 (n-3) was significantly reduced in the lipid phase of the insoluble protein fraction after US-treatment compared to control. There was also an increase (but not significant) in the amount of monounsaturated fatty acids (MUFAs) together with a reduction in the content of polyunsaturated fatty acids (PUFAs) in all US-treated samples compared to control. As it was mentioned earlier in sub-Chap. 3.1, this phenomenon can be explained by the increased emulsification of all US-treated samples compared to control due to a strong cavitation effect, resulting in trapping lipids by proteins, including PUFAs, and thus decreasing their content in insoluble protein fraction (Cropotova et al. 2024b). At the same time, the amount of saturated fatty acids (SFAs) in the lipid phase of insoluble protein fraction was not affected at all by US-treatment. Surprisingly, behenic acid (C22:0) was only present in small amounts in the fatty acid profile of control sample. Taking into account the fact that this fatty acid has a low bioavailability due to its very long chain length and is also proved to be a cholesterol-raising fatty acid, its presence in the fatty acid composition is not considered beneficial (Cater and Denke 2001). At the same time, US-treatment did not increase the amount of other long-chain saturated fatty acid C20:0 (arachidic acid) associated with increased levels of low-density lipoprotein (LDL) cholesterol and higher risk of cardiovascular disease, diabetes and metabolic syndrome (Siri-Tarino et al. 2010). Therefore, we may suggest that US-treatment had a positive effect on the fatty acid composition of insoluble protein fractions. The levels of other fatty acids were consistent across all samples at approximately 2–5%. Despite the significant differences observed in the amount of certain fatty acids among the samples, these changes cannot be considered substantial enough to be attributed to the effect of US-treatment. Moreover, although PUFAs are highly susceptible to oxidation, we cannot suggest this reason for the reduction of PUFAs in all US-treated samples, because TBARS results do not reflect significant changes in secondary lipid oxidation products (Table 3). Thus, we hypothesize that the differences in PUFA and MUFA content among the samples is attributed to variations in the fatty acid composition of the initial mackerel raw material used for hydrolysis.

**Table 2:** Fatty acid composition of the lipid part in insoluble protein fraction, reported as % of total fatty acids. Mean values ± standard deviation is shown. Different letters indicate significant differences (*p* < 0.05)

Fatty acid	Control	300 W	450 W	600 W
14:0	5.41 ± 0.02^a^	5.58 ± 0.02^ab^	5.61 ± 0.01^b^	5.58 ± 0.02^ab^
14:1	0.16 ± 0.12^a^	0.09 ± 0.04^ab^	0.03 ± 0.02^a^	0.07 ± 0.01^ab^
16:0	18.44 ± 0.03^a^	19.32 ± 0.02^b^	19.38 ± 0.02^b^	19.18 ± 0.00^b^
16:1 (n-7)	3.84 ± 0.02^ab^	3.69 ± 0.02^a^	4.01 ± 0.02^b^	3.99 ± 0.02^b^
18:0	4.15 ± 0.02^a^	4.36 ± 0.00^a^	4.42 ± 0.01^a^	4.23 ± 0.01^a^
18:1 (n-9)	15.86 ± 0.05^a^	16.21 ± 0.01^ab^	16.49 ± 0.00^ab^	17.38 ± 0.05^b^
18:1 (n-7)	3.21 ± 0.01^a^	3.12 ± 0.01^a^	3.33 ± 0.00^a^	3.34 ± 0.01^a^
18:2 (n-6)	1.47 ± 0.04^a^	1.51 ± 0.01^a^	1.44 ± 0.00^a^	1.48 ± 0.00^a^
18:3 (n-3)	1.19 ± 0.01^a^	1.15 ± 0.00^a^	1.14 ± 0.01^a^	1.19 ± 0.00^a^
18:4 (n-3)	2.75 ± 0.01^b^	2.59 ± 0.00^a^	2.53 ± 0.00^a^	2.62 ± 0.01^ab^
20:0	0.22 ± 0.01^a^	0.21 ± 0.00^a^	0.21 ± 0.01^a^	0.21 ± 0.01^a^
20:1 (n-9)	7.27 ± 0.06^a^	7.32 ± 0.02^ab^	7.58 ± 0.05^b^	7.62 ± 0.03^b^
20:2 (n-6)	0.25 ± 0.00^a^	0.27 ± 0.01^a^	0.26 ± 0.01^a^	0.26 ± 0.01^a^
20:4 (n-6)	0.67 ± 0.01^b^	0.66 ± 0.01^b^	0.63 ± 0.01^ab^	0.56 ± 0.01^a^
20:3 (n-3)	0.19 ± 0.01^a^	0.36 ± 0.12^b^	0.18 ± 0.00^a^	0.20 ± 0.01^a^
20:5 (n-3)	6.85 ± 0.04^b^	5.95 ± 0.01^ab^	6.08 ± 0.03^ab^	5.89 ± 0.04^a^
22:0	0.05 ± 0.08	-	-	-
22:1 (n11 + 13)	11.09 ± 0.15^a^	11.35 ± 0.05^ab^	11.54 ± 0.10^b^	11.41 ± 0.06^b^
22:1 (n-9)	0.99 ± 0.03^a^	1.02 ± 0.00^ab^	1.09 ± 0.02^b^	1.04 ± 0.01^ab^
22:5 (n-3)	1.31 ± 0.02^b^	1.19 ± 0.01^ab^	1.18 ± 0.01^ab^	1.13 ± 0.01^a^
22:6 (n-3)	13.42 ± 0.09^c^	12.62 ± 0.03^b^	11.59 ± 0.08^ab^	11.41 ± 0.08^a^
24:1	1.23 ± 0.01^a^	1.41 ± 0.01^b^	1.3 ± 0.01^ab^	1.22 ± 0.02^a^
MUFA^1^	43.64 ± 5.32^a^	44.2 ± 5.44^a^	45.37 ± 5.54^a^	46.07 ± 5.76^a^
PUFA^2^	28.1 ± 4.2^b^	26.3 ± 3.88^ab^	25.03 ± 3.61^ab^	24.74 ± 3.55^a^
SFA^3^	28.27 ± 6.69^a^	29.47 ± 7.01^a^	29.62 ± 7.03^a^	29.2 ± 6.96^a^
Lipid nutritional quality indexes				
Ratio ω-6:ω-3	1:10.2	1:9.3	1:9.2	1:9.3
AI^4^	0.56	0.59	0.59	0.59
TI^5^	0.25	0.29	0.29	0.29
HH^6^	1.68	1.55	1.52	1.55

^1^MUFA: monounsaturated fatty acids; ^2^PUFA: polyunsaturated fatty acids; ^3^SFA: saturated fatty acids; ^4^AI: atherogenicity index; ^5^TI: thrombogenicity index; ^6^HH: hypocholesterolemic fatty acid ratio

**Table 3:** Lipid and protein oxidation parameters in insoluble protein fraction. Mean value and standard deviation are shown. Different letters indicate significant differences (*p* < 0.05)

Parameters	Insoluble protein fractions after enzymatic hydrolysis			
	Control	300 W	450 W	600 W
TBARS, MDA/ kg oil	5.23 ± 0.29^a^	5.35 ± 0.34^a^	5.66 ± 0.44^a^	5.42 ± 0.42^a^
Thiol groups in water-soluble proteins, nmol/mg protein	1.48 ± 0.14^a^	4.28 ± 0.91^b^	2.08 ± 0.09^c^	0.47 ± 0.02^d^
Thiol groups in salt-soluble proteins, nmol/mg protein	2.41 ± 0.26^a^	1.65 ± 0.36^b^	1.83 ± 0.26^b^	0.19 ± 0.06^c^

### Nutritional quality of the lipid part

The nutritional quality of the lipids found in the insoluble protein fraction was be assessed by the ratio of ω-6 to ω-3 fatty acids (Pigott and Tucker 1990; Simopoulos 2002), as well as atherogenicity index (AI), thrombogenicity index (TI), and hypocholesterolemic fatty acid ratio index (HH) (Dave et al. 2024). The recommended ratio of ω-6 to ω-3 fatty acids in human nutrition is lower than 5:1 (Ruxton et al. 2004; Mai et al. 2023). In our study, the ratios of ω-6 to ω-3 fatty acids in the lipids recovered from the insoluble protein fractions were significantly higher than those recommended and constituted 1:10 for control sample and 1:9 for experimental samples obtained after US-treatment. A lower ratio between these fatty acids is generally preferred, as it is considered to be healthier in terms of reduction of inflammatory processes in the organism (Simopoulos 2002). In our study, the control sample of the lipids has the lowest ω-6 to ω-3 ratio, thus implying potentially healthier inflammatory responses. However, US-treated samples also have quite good balance between these two types of fatty acids. The obtained results suggest that the lipid phase of the insoluble protein fraction has a nutritionally favourable fatty acid composition for mammal nutrition and can be used in animal feed.

The AI has been widely used for assessing the atherogenic potential and nutritional quality of lipid-rich foods, including marine raw material (Chen and Liu 2020). According to Akintola (2015), the AI of sun-dried and smoked southern pink shrimp (*Penaeus notialis*) varied between 0,71 and 0,82. For fish this index is normally found in the rage of 0,21 − 1,41 (Fernandes et al. 2014; Tonial et al. 2014; Łuczyńska et al. 2017; Chen and Liu 2020). In our study, AI of control sample of insoluble protein fraction was 0,56 (Table 2), while AI of all three experimental samples subjected to US-treatment was 0,59. Despite the slight increase of AI after US-treatment, all insoluble proteins fractions had low values of AI corresponding to the AI range of 0,26 − 0,33 for pelagic fish species found in previous studies (Fernandes et al. 2014; Tonial et al. 2014; Łuczyńska et al. 2017; Chen and Liu 2020). This fact suggests that all insoluble proteins fractions obtained after enzymatic hydrolysis of mackerel side streams can reduce the levels of total cholesterol and LDL-C in blood plasma (Chen and Liu 2020).

TI has been largely used for evaluation of the degree of thrombogenicity in many food products based on their fatty acid composition (Chen and Liu 2020). This nutritional index shows the tendency to form clots in the blood vessels (Ghaeni and Ghahfarokhi 2013). In the present study, TI of the control sample was 0,25 (Table 4). However, US-treatment increased the values of TI for all insoluble protein fractions up to 0,29. Nevertheless, all insoluble protein fractions had quite low TI values found in the range of thrombogenicity index 0,21 − 0,33 characteristic for shrimp (Akintola 2015) and fish raw material (Fernandes et al. 2014; Tonial et al. 2014; Łuczyńska et al. 2017; Zula et al. 2021). Thus, all insoluble protein fractions obtained after enzymatic hydrolysis of mackerel side streams can be considered beneficial to support cardiovascular health (Chen and Liu 2020).

**Table 4:** Amino acid composition of insoluble protein fraction. Mean values ± standard deviation is shown (no significant differences Amoung the treatments at *p* < 0.05)

Amino acids (mg/g)	Control	300 W	450 W	600 W
Aspartic acid	47.79 ± 9.63	50.15 ± 9.23	44.36 ± 8.08	41.25 ± 15.53
Glutamic acid	56.59 ± 11.94	60.02 ± 11.60	52.60 ± 10.20	50.28 ± 19.26
Asparagine	0.05 ± 0.02	0.00 ± 0.01	0.02 ± 0.00	0.02 ± 0.02
Histidine	14.38 ± 2.24	8.94 ± 4.22	11.02 ± 2.35	11.51 ± 4.24
Serine	16.41 ± 4.00	18.91 ± 4.10	15.92 ± 3.21	14.50 ± 5.92
Glutamine	0.32 ± 0.05	0.37 ± 0.10	0.43 ± 0.07	0.23 ± 0.06
Glycine/Arginine	60.47 ± 15.25	68.26 ± 14.36	62.22 ± 11.92	53.32 ± 24.77
Threonine	21.75 ± 4.45	23.65 ± 5.39	19.24 ± 3.74	19.14 ± 7.67
Alanine	27.08 ± 5.93	28.60 ± 5.59	25.56 ± 4.73	22.78 ± 9.43
Tyrosine	14.96 ± 4.66	17.49 ± 4.50	14.99 ± 4.38	14.62 ± 5.96
Aminobutyric acid	0.55 ± 0.60	0.60 ± 0.32	0.47 ± 0.17	0.49 ± 0.30
Methionine	14.35 ± 3.07	15.87 ± 2.82	13.49 ± 2.98	13.30 ± 5.33
Valine	29.65 ± 5.53	30.90 ± 4.94	27.40 ± 4.96	24.66 ± 9.52
Phenylalanine	25.07 ± 4.81	27.18 ± 4.90	23.88 ± 4.66	21.05 ± 8.93
Isoleucine	26.43 ± 4.73	28.10 ± 4.50	24.86 ± 4.67	22.91 ± 8.35
Leucine	40.00 ± 7.94	42.63 ± 7.62	37.50 ± 7.26	34.35 ± 12.88
Lysine	36.38 ± 8.27	41.40 ± 7.87	35.49 ± 6.76	33.48 ± 13.31
$\:{\Sigma\:}$ESAAs^1^	208.47 ± 15.55	218.67 ± 15.61	192.88 ± 13.96	180.40 ± 26.24
$\:{\Sigma\:}$NESAAs^2^	224.20 ± 23.26	244.40 ± 22.23	212.86 ± 19.06	197.50 ± 37.22
$\:{\Sigma\:}$TAAs^3^	432.67 ± 17.12	463.07 ± 27.16	405.74 ± 23.63	377.90 ± 45.54
Hydrophobic^4^	159.71 ± 14.33	173.28 ± 13.64	127.13 ± 13.08	116.27 ± 23.64

^1^ESAAs: essential amino acids; ^2^NESAAs: non-essential amino acids; ^3^TAAs: total amino acids; ^4^Hydrophobic: sum of alanine, tyrosine, methionine, valine, phenylalanine, isoleucine, and leucine

HH is a relatively new nutritional quality index to assess the effect of fatty acid composition composition on cholesterol (Chen and Liu 2020). However, a big variety of marine products have already been nutritionally characterized by using this index (Dal Bosco et al. 2013; Fernandes et al. 2014; Tonial et al. 2014; Paiva et al. 2016; Rincón-Cervera et al. 2020). Thus, Paiva et al. (2016) determined HH in four Azorean macroalgae species to assess their nutritional and health promoting properties, revealing that the HH value ranges from 1,26 to 2,09. For fish products, the HH values are normally ranging from 1,54 to 4,83 (Dal Bosco et al. 2013; Tonial et al. 2014; Rincón-Cervera et al. 2020). In the present study, HH was significantly higher for control sample (1,68) compared to experimental samples of insoluble protein fraction (1,52 − 1,55) after US-treatment (Table 2). This fact denotes a positive effect of ultrasonication before enzymatic hydrolysis of fish raw material on fatty acid composition of resulted insoluble protein fraction contributing to healthy cholesterol metabolism.

### NMR profiles of lipids in the insoluble protein fraction

The ^31^P NMR results showed that the phospholipids (PLs) accounted for 13 ± 1% of the lipid extract (Table 5). The peak from PC/PC-ether were the dominating peak in the ^31^P NMR spectra. Distinct peaks corresponding to PI, LPC and PE- ether were also clearly identified, while peaks from PE appeared in a crowded region of the spectra, making unambiguous identification difficult (and is thereby labelled as * and may include peaks from API and others PLs). There was no clear effect of US-treatment on the phospholipid content in the insoluble protein fractions. Similarly, the relative content of different phospholipid species was consistent across the treatments. PC (and PC ether) accounted for ca. 70–74% (weight% of total PLs) followed by PE* (13–16%), LPC (5–6%) and PI (5–7%).

**Table 5:** Results from 31P- NMR. Relative content of different phospholipid species, reported as weight% of total phospholipids. Mean value and standard deviation are shown (*n* = 2)

Phospholipid species	Control	300 W	450 W	600 W
^1^PC + PC ether	73.9 ± 0.1	74.7 ± 1.1	73.7 ± 2.6	70.5 ± 0.5
^2^PI	5.3 ± 0.1	5.2 ± 1.0	6.3 ± 0.5	6.8 ± 0.9
^3^LPC	5.6 ± 0.9	5.7 ± 0.3	6.0 ± 1.3	6.4 ± 0.5
^4^PE*	15.2 ± 0.9	14.5 ± 0.2	13.9 ± 0.8	16.3 ± 1.0

^1^PC: phosphatidylcholine; ^2^PI: phosphatidylinositol; ^3^LPC: lysophosphatidylcholine; ^4^PE: phosphatidylethanolamine

*PE peak was partly overlapped by other peaks

**Fig. 3:**
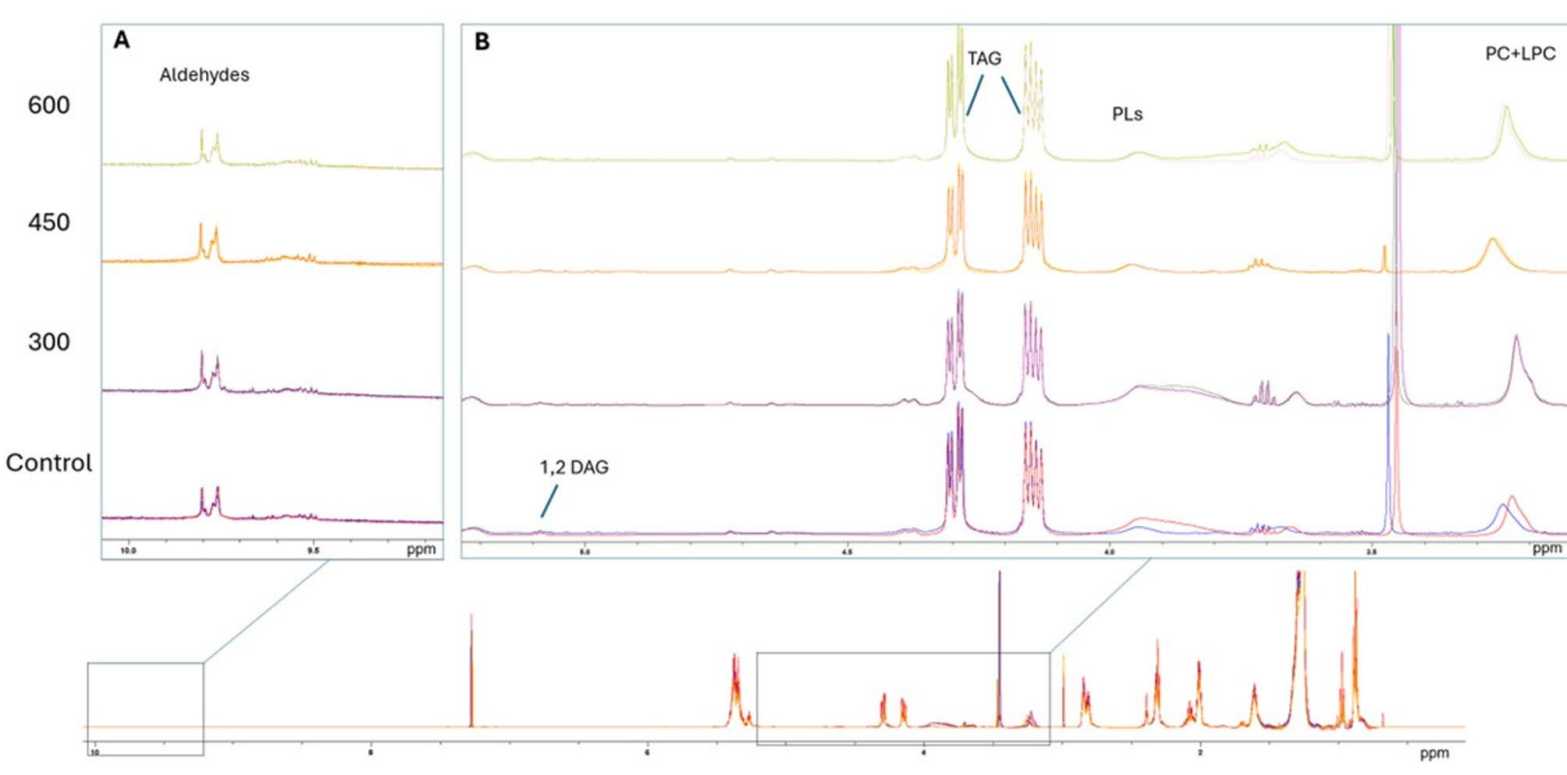
^1^H NMR spectra of lipid extracted from insoluble protein fraction. 3 A) carbonyl region (9–10 ppm) where aldehydes give signal, and 3B) glyceryl region (3.5–5.5 ppm), where information on lipid classes can be shown

From the ^1^H NMR glyceryl region (Fig. 3A), peaks from the lipid classes TAG, and 1,2 DAG were identified, in addition to broad peaks from PLs. There were no clear differences in the ^1^H NMR profiles due to the US treatment. In average, TAG accounted for 86 ± 4% mole of total fatty acids, and 1,2 diacylglycerols of 2.0 ± 0.5% of total FAs. Distinct peaks corresponding to aldehydes were observed in all samples, as shown in Fig. 3B. The aldehyde profile of oxidized marine lipids is more complex than that of vegetable oils, and a detailed identification of specific aldehydes was beyond the scope of this study. However, the intensity and overall profile of aldehyde peaks were similar across the samples (see further discussion on oxidative quality of oil in Chap. 3.8).

### Amino acid composition in the insoluble protein fraction

According to the total amino acid profile results displayed in Table 4, the most abundant amino acids in insoluble protein fractions were Glycine/Arginine, Glutamic acid, Aspartic acid and Leucine. These results are in accordance with our previous data from the study on US-treatment of mackerel FPH (Cropotova et al. 2024b). In this study, fish raw material contained high amounts of fish skin, and the most abundant amino acid was also Glycine followed by Glutamic acid (Cropotova et al. 2024b). Glycine and Arginine belong to the group of conditionally essential amino acids which may become essential in times of illness and stress (Trumbo et al. 2002). Glycine, as a major component of collagen, participates actively in the synthesis of skin cells and bone tissue, supporting skin and connective tissue health. Arginine takes part in the production of nitric oxide and enhances the immune function of T-cells, as well as collagen synthesis and cell proliferation (Trumbo et al. 2002). Therefore, the presence of these amino acids in abundance in the amino acid profile of insoluble protein fraction represents a positive factor from the nutritional point of view.

However, in the present study, there was no significant difference in amino acid distribution between the treatments (Table 4), suggesting that US-treatment doesn’t affect amino acid profile of insoluble protein fraction.

### Lipid and protein oxidation parameters

Lipid oxidation was determined in the oil phase of the insoluble protein fractions by measuring secondary lipid oxidation products as thiobarbituric acid reactive substances (TBARS). According to the results displayed in Table 3, there was no significant difference (*p* < 0.05) in TBARS-values between the samples, suggesting that ultrasound treatment before enzymatic hydrolysis did not affect their oxidative stability, which is in line with results from ^1^H NMR. This fact is in agreement with other studies investigating the effect of ultrasound treatment on lipid oxidation in fish products (Li et al. 2022a, b).

Protein oxidation taking place in the insoluble protein fractions was determined in terms of thiol groups in both water-soluble (sarcoplasmic) and salt-soluble (myofibrillar) proteins. Thiol groups of proteins are normally oxidized to disulfide bonds, resulting in a decrease in T-SH content (Higuera-Barraza et al. 2017). Intermolecular covalent disulfide bond reactions occur as a result of dehydrogenation of the SH groups of two cysteine residues in a protein molecule, producing oligomers which could further lead to aggregation and precipitation of proteins (Trivedi et al. 2009). Therefore, the formation of disulfide bonds is strongly associated with the reduction in protein solubility and functional properties of hydrolyzed protein (Cropotova et al. 2024a, b). According to Table 3, the insoluble protein fraction samples obtained after US-treatment at 300 W and 450 W before enzymatic hydrolysis, had significantly (*p* < 0,05) higher T-SH content in water-soluble proteins compared to control. According to the results of previous investigations (Wu et al. 2024), we hypothesize that the increased amount of total thiols in our samples subjected to ultrasonication, can be attributed to the unfolding of protein and breakage of disulfide bonds formed during US-treatment by enzymatic hydrolysis (Ali et al., 2023), thus forming total free thiol groups. However, total thiols decreased significantly (*p* < 0.05) in water-soluble proteins after US-treatment of 600 W applied to raw material before enzymatic hydrolysis compared to control. Taking into account the fact, that there was no significant difference in the secondary lipid oxidation products expressed by TBARS between the samples, this phenomenon can be explained by the intensified decomposition of water molecules during the high-power US-treatment with the formation of free radicals, which further decompose into hydrogen peroxide and oxidize thiol groups of sarcoplasmic proteins to disulfide bonds (Li et al. 2023). Regarding the oxidation of salt-soluble proteins, a significant (*p* < 0,05) decrease in total thiols was detected in all US-treated insoluble protein fractions compared to control. In accordance with the study of Shi et al. (2019) revealing a substantial decrease in total thiol content in myofibrillar proteins of grass carp (*Ctenopharyngodon idella*) after US-treatment, we suggest that this decrease is probably related to an irreversible oxidation of some of the thiol content in salt-soluble proteins producing sulfonic acids (-SO_3_H) (Sireesha et al. 2022). Thus, myofibrillar protein thiols in experimental insoluble fractions underwent redox alteration after being exposed to reactive oxygen species generated during ultrasonication. Due to the stronger cavitation effect of US-treatment increasing along with the increased power from 300 W to 600 W, more free radicals were released out of the cells (Soladoye et al. 2015), leading to a bigger decrease in total thiols (Table 3) due to higher oxidation of salt-soluble proteins. One of the main reasons for higher oxidation parameters of salt-soluble proteins in the present study, is their higher exposure to reactive oxygen species (ROS) during processing, including ultrasound pre-treatment and enzymatic hydrolysis (Qian et al. 2023). In addition, salt-soluble proteins often have exposed amino acid residues (e.g. cysteine, methionine, tryptophan) that are highly susceptible to oxidation, while water-soluble proteins tend to have these residues buried within their structure (Park and Chin 2011).

### Color parameters

According to the results displaying color parameters of the insoluble protein fraction in Table 6, no significant (*p* < 0.05) differences in lightness (L*-value) and redness (a*-value) between control sample and insoluble protein fractions obtained after US-treatment at 300 W and 450 W, were found. However, the lightness of the sample 600 W increased significantly compared to control. According to our hypothesis supported by other studies (Du et al. 2022; Cropotova et al. 2024a), the cavitation effect generated during high power ultrasound treatment at 600 W modified the secondary structure of proteins, shifting their light absorption peaks to higher wavelengths. At the same time, it was a significant decrease in redness of the sample 600 W compared to control, which can be attributed to ultrasonic denaturation of myosin (the main protein of myofibrillar protein in mackerel mince), leading to its cross-linking (Li et al. 2022a, b; Cropotova et al. 2024b). In addition, the significant decline in a*-value of this sample compared to control can be explained by oxidation of myoglobin (bright red) to metmyoglobin (reddish-brown) in the fish mince during ultrasonication (Sun et al. 2021). This hypothesis is strongly supported by the significant decline in total thiols in the sample 600 W compared to other samples and control (Table 3), suggesting the US-promoted oxidation of the red pigment during high-power ultrasonication of mackerel mince. According to the previous study of Sun et al. (2021), high-power ultrasound treatment is able to decompose water to produce free radicals, which may further promote the oxidation of fish proteins including muscle pigments. In addition, the generated cavitation effect may loosen bonds between fish muscle fiber proteins, thus promoting the entry of water into the fish raw material and dissolution of muscle pigments (Li et al. 2022a, b). Ultrasound-promoted loosening the fish myofibril network results in the formation of channels in the muscle structure, affecting its integrity and promoting myoglobin dissolution. This further leads to a significant decrease in myoglobin content in the insoluble protein fraction, which is consistent with the results of a*-value for the sample 600 W in our study. However, there was found no significant difference in yellowness (b*-value) between the insoluble protein fraction samples, which normally is attributed to lipid oxidation reactions (Sun et al. 2021; Li et al. 2022a, b; Cropotova et al. 2024b). These findings are in agreement with TBARS-values of the lipid phase of the insoluble protein fraction samples ascribed to secondary lipid oxidation products (Table 4). As in the case of b*-values, there was no significant difference in TBARS-values among the samples. In addition, a significant correlation (R^2^ = 0.99, *p* < 0.05) was found between yellowness and secondary lipid oxidation products expressed as TBARS for all insoluble protein fraction samples in the study.

**Table 6:** Color parameters of mackerel insoluble protein fraction after enzymatic hydrolysis. Mean values ± standard deviation is shown. Different letters in each row indicate significant differences between the samples (*p* < 0.05)

	Control	300 W	450 W	600 W
L*-value	41.68 ± 0.35^a^	41.13 ± 0.50^a^	42.50 ± 0.47^a^	50.59 ± 0.92^b^
a*-value	5.40 ± 0.29^a^	5.86 ± 0.50^a^	5.45 ± 0.21^a^	3.72 ± 0.16^b^
b*-value	10.81 ± 0.35^a^	10.27 ± 2.38^a^	9.32 ± 1.30^a^	10.09 ± 1.64^a^

## Conclusion

The study investigated the potential of ultrasound (US) pre-treatment to enhance the nutritional quality and functional properties of insoluble protein fractions obtained after enzymatic hydrolysis of Atlantic mackerel side streams. The obtained results show that US pre-treatment at the ultrasonication intensities of 450 W and 600 W can significantly (*p* < 0,05) increase solubility of both water-soluble (sarcoplasmic) and salt-soluble (myofibrillar) proteins. This improvement is attributed to the cavitation effect of ultrasonication, which breaks down protein bonds and exposes hydrophilic groups, thus increasing protein solubility. The cavitation effect resulted also in increased degree of hydrolysis for the sample 450 W.

Moreover, protein oxidation expressed as a reduction in total thiols in both water- and salt-soluble proteins, was notably influenced by US pre-treatment. Thus, a significant decline in total thiols was observed in all salt-soluble proteins of the insoluble protein fractions after US-treatment, while for water-soluble proteins the negative changes were only observed at US pre-treatment at 450 W compared to control. Despite these changes, secondary lipid oxidation products expressed as TBARS, and aldehyde profiles mapped by ^1^H NMR, remained unaffected by US pre-treatment across all samples. Moreover, US-treated fractions exhibited a favourable nutritional quality in terms of low IA and IT nutritional indexes and improved hypocholesterolemic profiles in terms of HH index compared to control, suggesting improved effect on healthy cholesterol metabolism.

The dominating lipid class in the insoluble fraction was TAG, followed by PLs. ^31^P-NMR profile of the extracted lipids revealed that the most abundant phospholipid species were PC and PC-ether accounting for ca. 70–74% of the total PL content. However, the lipid class composition and relative content of different phospholipid species appeared similar across the treatments.

In addition, there was no significant differences in colour parameters (L*-value, a*-value and b*-value) among the samples of insoluble protein fractions, except for the sample obtained after US-treatment at 600 W. The increased lightness (L*-value) and reduced redness (a*-value) of this sample is probably attributed to the stronger cavitation effect generated during high power ultrasound treatment at 600 W, resulting in oxidation of myoglobin and modification of the secondary structure of proteins.

To conclude, based on total protein content, PUFA content, lipid nutritional quality indexes, and protein oxidation parameters of insoluble protein fraction, US-pre-treatment at 300 W before enzymatic hydrolysis is more promising than US-treatments at 450 W and 600 W.

## Data Availability

The datasets used in the current study are available from the corresponding author on reasonable request.

## Abbreviations

a*: Redness
AI: Atherogenicity index
b*: Yellowness
BSA: Bovine serum albumin
DH: Degree of hydrolysis
DHA: Docosahexaenoic acid
DTNB: 5,5-dithio-bis-(2-nitrobenzoic acid) (Ellman’s Reagent)
EPA: Eicosapentaenoic acid
ESAAs: Essential amino acids
ESFAAs: Essential free amino acids
FAA: Free amino acids
FFA: Free fatty acids
FPH: Fish protein hydrolysate
GC: Gas chromatography
HH: Hypocholesterolemic fatty acid ratio
^1^H NMR: Proton nuclear magnetic resonance
HPP: High-pressure processing
L*: Lightness
LPC: Lysophosphatidylcholine
MAE: Microwave-assisted extraction
MDA: Malondialdehyde
MUFA: Monounsaturated fatty acids
NESAAs: Non-essential amino acids
NESFAAs: Non-essential free amino acids
NTNU: Norwegian University of Science and Technology
P-NMR: Phosphorus-31 Nuclear Magnetic Resonance
PC: Phosphatidylcholine
PE: Phosphatidylethanolamine
PI: Phosphatidylinositol
PUFA: Polyunsaturated fatty acids
ROS: Reactive oxygen species
SFA: Saturated fatty acids
SFE: Supercritical fluid extraction
SH: Sulfhydryl
TAAs: Total amino acids
TAGs: Triacylglycerols
TBARS: Thiobarbituric acid reactive substances
TI: Thrombogenicity index
TFAAs: Total free amino acids
TMS: Trimethylsilane
US: Ultrasound
UV: Ultraviolet
